# 

**Published:** 1869-04

**Authors:** 


### CONTENTS.

ORIGINAL COMMUNICATIONS.

The Dental Profession *vs.* Rubber.......... 141
The Health of the Dentist—Means of Preserving.......... 147
When should the File be used?.......... 157
Some Remarks on Metal Plates.......... 160
Root Filling.......... 161
Correspondence.......... 162

SELECTIONS.

Difference of Excretion of Carbonic Acid and Absorption of Oxygen under various circumstances.......... 165
Diseases of the Jaws.......... 168
Notes from L'Union Medicale.......... 174
Tinting Vegetable Tissues.......... 176
Detection of Mercury in Cases of Poisoning.......... 176
Temperature of the Body in Health.......... 177
Influence of Medicaments on the Heart.......... 178
Assaying Silver Compounds in the Wet Way.......... 178
How to Count the Lines in Nobert's Plates.......... 179
The First Physician in Massachusetts.......... 179
Western New York Dental Association.......... 179
North Iowa Dental Association.......... 180

EDITORIAL.

"The Health of the Dentist".......... 181
Commencement.......... 182
Atlas to the Pathology of the Teeth.......... 183
The Mad River Dental Society.......... 184
Obituary.......... 184

